# Human Vascular Microphysiological System for *in vitro* Drug Screening

**DOI:** 10.1038/srep21579

**Published:** 2016-02-18

**Authors:** C. E. Fernandez, R. W. Yen, S. M. Perez, H. W. Bedell, T. J. Povsic, W. M. Reichert, G. A. Truskey

**Affiliations:** 1 Department of Biomedical Engineering, Duke University, Durham, NC 27708; 2 Duke Clinical Research Institute, Duke University Medical Center, Durham, NC 27708.

## Abstract

*In vitro* human tissue engineered human blood vessels (TEBV) that exhibit vasoactivity can be used to test human toxicity of pharmaceutical drug candidates prior to pre-clinical animal studies. TEBVs with 400–800 μM diameters were made by embedding human neonatal dermal fibroblasts or human bone marrow-derived mesenchymal stem cells in dense collagen gel. TEBVs were mechanically strong enough to allow endothelialization and perfusion at physiological shear stresses within 3 hours after fabrication. After 1 week of perfusion, TEBVs exhibited endothelial release of nitric oxide, phenylephrine-induced vasoconstriction, and acetylcholine-induced vasodilation, all of which were maintained up to 5 weeks in culture. Vasodilation was blocked with the addition of the nitric oxide synthase inhibitor L-N^G^-Nitroarginine methyl ester (L-NAME). TEBVs elicited reversible activation to acute inflammatory stimulation by TNF-α which had a transient effect upon acetylcholine-induced relaxation, and exhibited dose-dependent vasodilation in response to caffeine and theophylline. Treatment of TEBVs with 1 μM lovastatin for three days prior to addition of Tumor necrosis factor – α (TNF-α) blocked the injury response and maintained vasodilation. These results indicate the potential to develop a rapidly-producible, endothelialized TEBV for microphysiological systems capable of producing physiological responses to both pharmaceutical and immunological stimuli.

Currently, over 80% of proposed pharmaceutical drug candidates that enter clinical trials fail due to concerns with human efficacy and toxicity^1^. While pre-clinical animal studies provide great value, animal responses to drugs may exhibit differences in toxic doses and drug metabolism^2^. Microphysiological systems (MPS) are perfused small-scale models of one or more human tissues or organs^3^ comprised of human primary cells or induced pluripotent stem cells (iPSCs) with the ultimate potential of becoming models to study disease or tools for precision medicine. In order to accurately model disease and predict drug responses on an organ scale, three-dimensional (3D) human tissue models are critical. Many pre-clinical studies are conducted on two-dimensional (2D) plastic or glass substrates; however, *in vivo*, tissues are comprised of complex extracellular matrices embedded with mixed populations of cells and perfused with fluids.

In addition to facilitating more accurate disease models through interaction with immunological stimuli, human MPS may serve as a bridge in the drug development pipeline between 2D cell culture studies and *in vivo* animal studies. Assessment of toxicity within the vasculature is of particular importance, since drug-induced vascular injury (DIVI), which typically manifests in preclinical animal studies through inflammation and changes in vascular tone, precludes many drug candidates from continuing along the pipeline despite uncertain characterization of human DIVI response^4^. In humans, cancer chemotherapeutics cause vascular damage affecting flow-mediated vasodilation^5^,^6^. Three-dimensional (3D) tissue models have the potential to allow us to evaluate human biological interactions and diseases by taking advantage of natural spatiotemporal cues, physiological fluid perfusion, a variety of cell types, and the complex extracellular matrix that are present in tissues but are absent from 2D culture plates^7^. A human tissue-engineered blood vessel (TEBV) capable of responding to vasoreactive stimuli would pose a promising model for the evaluation and screening of pharmaceutical drug candidates for toxicity and efficacy within the circulatory system.

An ideal TEBV for MPS applications would be comprised of human cells in a biological or biodegradable synthetic matrix, have a small inner diameter to reduce fluid volumes, exhibit enough mechanical strength to withstand physiological stresses, and be produced rapidly to facilitate efficient drug screening. The medial wall cells should exhibit a smooth muscle phenotype, be quiescent and be able to contract and relax in response to agonists or inhibitors. Most importantly, the TEBV must be endothelialized to enable physiologically relevant dilation and constriction in response to stimuli.

TEBVs have been constructed using three general approaches: natural or biodegradable synthetic matrices populated *in vitro* with cells, self-assembled cell sheets, or *in vivo* repopulation of decellularized natural or synthetic vessel matrices^8^,^9^. Despite their biomimetic properties, the sizes and long culture times for fabrication of TEBVs by many of the current approaches creates challenges in applying these procedures to *in vitro* drug testing^10^. While TEBVs constructed from natural matrix components such as collagen^11^,^12^ and fibrin^13^,^14^ have traditionally exhibited poor mechanical strength, plastic compression of collagen gels embedded with smooth muscle cells increases the collagen fiber density and yields rapidly-producible tubular structures with high mechanical strength^15^.

A functional TEBV requires a confluent endothelial layer. The endothelium plays a major role in regulating leukocyte and platelet adhesion, permeability, and vascular tone, as well as modulating vasodilation through release of nitric oxide in response to changes in flow or stimuli with vasoreactive compounds such as acetylcholine^16^. Endothelial coverage of TEBVs prior to implantation in animal models has been assessed with characteristic endothelial markers such as von Willebrand factor (vWF)^17^,^18^. Under static *in vitro* conditions, endothelium seeded on TEBVs made from cultured SMC and fibroblast sheets demonstrated nearly confluent coverage, expressed vWF and inhibited platelet adhesion^10^. After exposure to physiological shear stresses for 24–48 hours, endothelial progenitor cells (EPCs) remain adherent to fibrin scaffolds embedded with either human neonatal dermal fibroblasts (hNDFs) or smooth muscle cells (SMCs), deposit collagen IV and laminin, and upregulate cell adhesion molecules VCAM-1 and ICAM-1 upon exposure to pro-inflammatory cytokines such as tumor necrosis factor-α (TNF-α)^19^,^20^.

Non-destructive evaluation of TEBV maturation and vasoactivity is beneficial toward effective monitoring of responses to drugs or stimuli. Table 1 summarizes previous vasoactivity assessments performed on TEBVs. Although TEBVs have been assessed for endothelium-independent vasoconstriction or vasodilation using a variety of agents, TEBVs comprised of human cells have yet to be evaluated for endothelium-dependent vasoactivity under physiological fluid perfusion conditions. One important clinical assessment of cardiovascular health, which predicts future cardiac events, involves infusion of acetylcholine to induce vasodilation of the coronary or brachial arteries^21^,^22^,^23^. Acetylcholine is a muscarinic cholinergic receptor agonist that stimulates release of nitric oxide, prostacyclin, and endothelium-derived hyperpolarizing factor in vessels with healthy, intact endothelium. Conversely, acetylcholine activates the muscarinic receptors on the smooth muscle cells in vessels with dysfunctional or damaged endothelium, leading to vasoconstriction^21^,^24^.

In this study, dense collagen gels^15^,^25^ embedded with human neonatal dermal fibroblasts (hNDFs) or human bone marrow-derived mesenchymal stem cells (hMSCs) were used to construct TEBVs that can be perfused under physiological conditions in a few hours. We compared hNDFs to hMSCs as a medial cell source to determine the cell source that would provide the greatest mechanical properties and contractility within 1–2 weeks. TEBV lumens were endothelialized with endothelial progenitor cells derived from patients with coronary artery disease (CAD EPCs)^26^,^27^. We used CAD EPCs because they express markers of healthy endothelium and function similar to human aortic ECs^27^, and provide the potential for future studies of a population pre-disposed to atherosclerosis. Perfused and endothelialized TEBVs exhibited high mechanical strength and contractility after one week and maintained these properties for five weeks *in vitro*. Phenylephrine and acetylcholine were used to non-destructively measure endothelium-independent vasoconstriction and endothelium-dependent vasodilation, respectively. We have used the non-specific phosphodiesterase inhibitors caffeine and theophylline to evaluate drug-induced vasodilation. We further evaluated endothelial function by measuring nitric oxide production and the acute inflammatory response following exposure to TNF-α. Finally, we have evaluated the potential for drug response testing through exposure to lovastatin for 3 days prior to exposure to TNF-α.

## Results

### Generation of Small-Diameter, Endothelialized TEBVs

**Figure 1:**
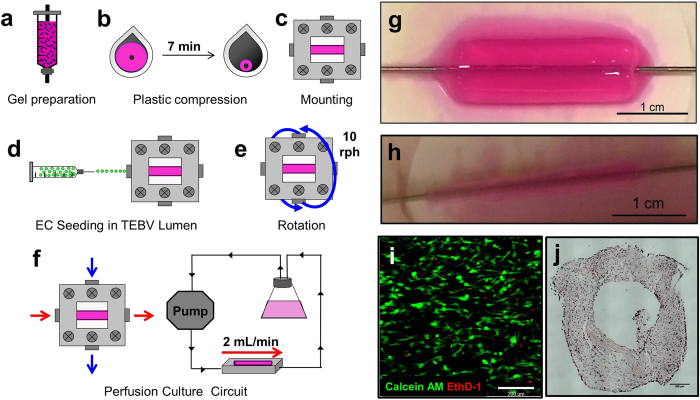
TEBV Fabrication and Culture. A suspension of hNDFs in collagen is poured into the mold containing an 810-μm diameter mandrel and allowed to gel for 30 minutes (**a**). Collagen fiber density increases through plastic compression and removal of water (**b**). Compressed TEBVs are immediately mounted in custom chambers (**c**). CAD EPCs are seeded into the lumen of the TEBV (**d**) and the chamber is rotated at 10 rph for 30 minutes (**e**). After endothelialization, TEBVs are mounted into the perfusion circuit and cultured for at least 1 week at a flow rate of 2 mL/min (**f**). TEBVs before (**g**) and after compression (**h**). Live-dead assay performed 24 hours after compression (**i**). H&E cross-section of TEBV after 1 week of perfusion culture (**j**). Scale bars indicate 200 μm unless otherwise noted.

TEBVs with dense collagen gel matrices were constructed by first embedding human neonatal dermal fibroblasts (hNDFs) or mesenchymal stem cells (hMSCs) in rat-tail collagen I matrices^15^,^25^. The hNDF-collagen I mixture was poured into a 3-cc syringe (BD Biosciences) containing a concentric stainless steel mandrel 810 μm in diameter and allowed to gel for 30 minutes (Fig. 1a). Afterward, TEBVs were plastically compressed by suspension on absorbent tissue paper (Fig. 1b) which reduced water volume and increased collagen fiber density (Table S1).

After compression, TEBVs were immediately placed in warmed media and mounted onto custom-made perfusion bioreactor chambers (Fig. 1c). To create an endothelial layer, 500,000 CAD EPCs were injected into the TEBV lumen (Fig. 1d), mounted on a custom rotation platform, and rotated at 10 rph for 30 minutes at 37 °C (Fig. 1e). Endothelialized TEBVs were immediately perfused at 2 mL/min to produce an initial laminar shear stress of 6.8 dynes/cm^2^. Time-averaged arterial wall shear stress ranges from 5–12 dynes/cm^2^ in healthy adult humans^28^. TEBVs were perfused for 1–5 weeks to evaluate maturation and vasoactivity over time (Fig. 1f). Plastic compression substantially reduced TEBV outer diameter (Fig. 1g,h). A live-dead assay performed on TEBVs 24 hours after compression indicates cell survival after compression (Fig. 1i). TEBVs cultured for one week demonstrated a uniform distribution of medial cells throughout the vessel wall (Fig. 1j).

### Mechanical strength and stability of TEBVs

**Figure 2:**
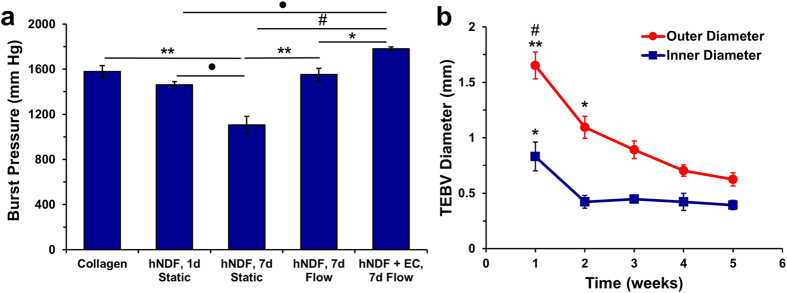
TEBV Mechanical Properties and Stability. TEBVs comprised of collagen only maintained under static conditions for 24 hours demonstrated burst pressures of 1577.6 ± 54.5 mm Hg. TEBVs embedded with hNDFs and maintained under static conditions for 24 hours after preparation exhibited burst pressures of 1460.1 ± 28.8 mm Hg, but 1 week of culture under static conditions led to burst pressures of 1104.5 ± 77.1 mm Hg. Exposure to flow increased the mechanical properties of the TEBVs after 7 days, leading to burst pressures of 1552.6 ± 54.9 mm Hg. Endothelialized TEBVs perfused for 7 days yielded burst pressures of 1777.6 ± 15.8 mm Hg Values are reported as mean ± SEM, n = 3, 4 TEBVs. (*p < 0.05, ^●^p < 0.01, **p < 0.005, ^#^p < 0.0001) (**a**). A one-way repeated measures ANOVA and the post-hoc Tukey test indicated that the inner diameter of endothelialized hNDF TEBVs stabilized after the second week in culture (*P < 0.05 with respect to weeks 2–5). The outer diameter stabilizes after the third week in culture (*P < 0.05 with respect to week 5; **P < 0.005 with respect to weeks 2 and 3; ^#^P < 0.0001 with respect to weeks 4 and 5.) Values are mean ± SEM. Four independent experiments were conducted. (**b**).

Burst pressure was measured by pressurizing TEBVs with PBS until failure^29^, and circumferential strength of TEBV slices was measured using a tensile testing apparatus with modified grips as previously described^15^,^30^. TEBVs comprised of collagen only maintained in PBS for 24 hours initially exhibited high burst pressure and tensile strength (Fig. 2a, Fig. S1). Burst pressures decreased slightly after introduction of hNDFs into the scaffold for 24 hours, and dropped substantially after 1 week under static conditions. Mechanical properties increased with mechanical stimulation. After one week, hNDF TEBVs matured under perfusion demonstrated significantly higher burst pressures compared to those under static conditions (p < 0.005). Endothelialized TEBVs matured for one week under physiological perfusion demonstrated the greatest burst pressures. After 1 week, perfused, endothelialized TEBVs exhibited greater tensile stress and Young’s modulus (Fig. S1).

TEBV wall thickness was assessed by measuring inner and outer diameters at one-week intervals over a 5-week perfusion period as described in Materials and Methods. Both the inner and outer diameter declined between the first and second week of perfusion, and stabilized by the third week (Fig. 2b). The pressure drop across the lumen of the TEBVs ranged from 0.5 mmHg – 5 mmHg at flow rates of 1.5–4 mL/min.

### Contractile and Extracellular Matrix Protein Expression

**Figure 3:**
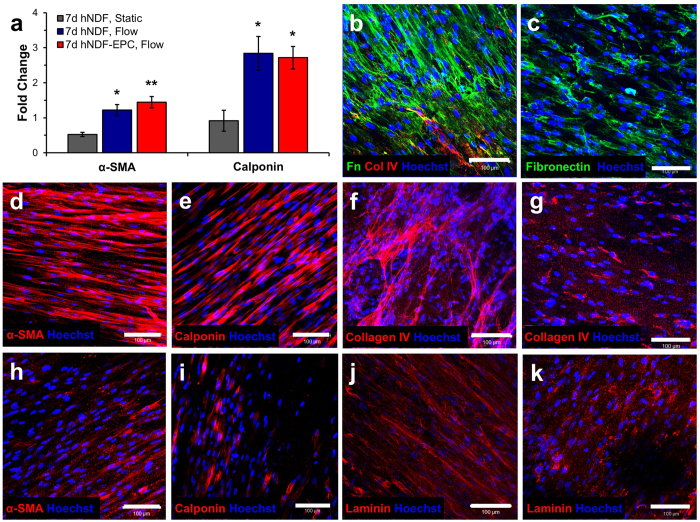
Expression of contractile and extracellular matrix proteins in endothelialized TEBVs. Perfusion for 1 week increased mRNA expression of α-SMA and calponin compared to TEBVs under static conditions. One-way ANOVAs with post-hoc Tukey’s tests were performed on the results of four independent experiments performed with hNDF-only TEBVs and hNDF TEBVs under flow and three independent experiments performed with endothelialized TEBVs matured for 1 week under physiological perfusion. (*p < 0.05, **p < 0.005; mean ± SEM). Calponin results were normalized to reference RNA derived from a TEBV matured under static conditions for 24 hours (**a**). Endothelialized TEBVs composed of hNDFs demonstrated greater expression of contractile proteins α-SMA and calponin after 1 week in perfusion culture (**d**,**e**), compared to endothelialized TEBVs composed of hMSCs (**h**,**i**). After 1 week of perfusion culture, endothelialized TEBVs composed of hNDFs produced more basement membrane proteins fibronectin (**b**), collagen IV (**f**), and laminin (**j**) than endothelialized TEBVs composed of hMSCs (**c**,**g**,**k**), respectively. All scale bars represent 100 μm.

At fabrication, hNDFs in TEBVs expressed low levels of α-SMA (Figure S2). Mechanical stimulation increased the expression of mRNA specific to the contractile proteins α-smooth muscle actin (α-SMA) and calponin in hNDFs (Fig. 3a). After 1 week of perfusion, hNDFs expressed α-SMA and calponin (Fig. 3d,e), indicating differentiation toward a myofibroblast phenotype^31^. Endothelialized TEBVs composed of hMSCs also expressed α-SMA and calponin after 1 week; however, to a lesser extent than hNDFs (Fig. 3h,i). TEBVs perfused for up to 5 weeks at a laminar flow rate of 2 mL/minute maintained production of α-SMA and calponin throughout this period (Fig. S3). Fibronectin expression was evaluated as a marker of TEBV remodeling^32^. Endothelialized hNDF TEBVs demonstrated greater fibronectin expression compared to endothelialized hMSC TEBVs (Fig. 3b,c). TEBVs made with hNDFs and hMSCs expressed endothelial basement membrane proteins collagen IV (Fig. 3f,g), and laminin (Fig. 3j,k) after 1 week. Thus, after 1 week of perfusion, TEBVs composed of hNDFs are more phenotypically mature compared to hMSCs.

### Vasoactivity

**Figure 4:**
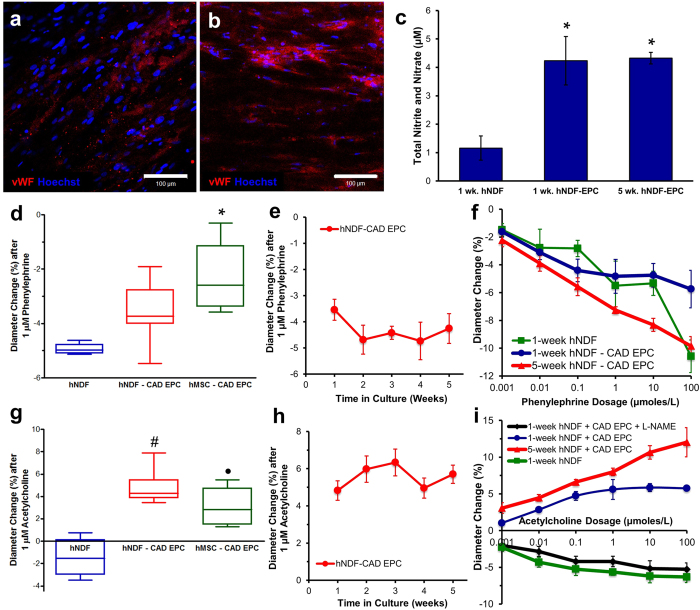
TEBVs maintain endothelial coverage, nitric oxide (NO) production, and vasoactivity during 5 weeks of perfusion. Endothelialized TEBVs expressed vWF after 1 (**a**) and 5 weeks (**b**) and produced NO at similar rates after 1 and 5 weeks of perfusion (**c**). Endothelialized TEBVs elicit significantly more NO compared to TEBVs matured for one week without endothelium. Nitrates and nitrites were sampled from media perfused through TEBV for 48 hours and values represent mean ± SEM. NO from six hNDF-only TEBVs was measured in three independent experiments (N = 3) and from four independently cultured TEBVs measured at 1 and 5 weeks (N = 4). Four separate CAD EPC donors were evaluated and pooled. A one-way ANOVA with a post-hoc Tukey’s test indicates a significant effect of treatment on NO production (*P < 0.05), with respect to TEBVs without endothelium cultured for 1 week under perfusion (**c**). Endothelialized TEBVs elicit greater vasoconstriction to 1 μM phenylephrine after one week compared to endothelialized TEBVs with hNDFs and hMSCs (*P < 0.05 with respect to hNDF) (**d**). Endothelialized TEBVs composed of hNDFs maintain consistent vasoconstriction to 1 μM phenylephrine during 5 weeks of perfusion (**e**). A one-way repeated measures ANOVA indicated a significant effect of phenylephrine dose (P < 0.05, **f**). Endothelialized TEBVs composed of hNDFs or hMSCs elicited vasodilation to acetylcholine, compared to TEBVs without endothelium which constricted (^●^P < 0.01 and ^#^P < 0.0001 compared to non-endothelialized hNDFs) (**g**). Endothelialized hNDF TEBVs maintained consistent vasodilation to acetylcholine over 5 weeks of perfusion (**h**). Endothelialized hNDF TEBVs perfused for 5 weeks elicited greater vasodilation in response to acetylcholine compared to endothelialized TEBVs perfused for 1 week. TEBVs cultured for 1 week without endothelium elicit increasing vasoconstriction in response to increasing doses of acetylcholine until 1 μM. TEBVs pre-treated with L-NAME exhibit constriction in response to increasing doses of acetylcholine (N = 4). Four hNDF-only TEBVs, four endothelialized TEBVs cultured for 1 week, and three endothelialized TEBVs cultured for 5 weeks were analyzed. A one-way repeated measures ANOVA confirmed a significant effect of acetylcholine dose (P < 0.0001). (**i**).

Endothelial presence was verified by staining the lumen of the TEBVs for von Willebrand factor (vWF), which is expressed in the Weibel-Palade bodies of healthy endothelium. Endothelialized TEBVs cultured for 1 week and 5 weeks both expressed vWF (Fig. 4a,b, respectively). Isotype matched controls indicated no background staining of endothelium. Nitric oxide in the culture medium was evaluated by assessing total nitrite and nitrate, the stable byproducts of nitric oxide metabolism (Fig. 4c). NO production of endothelialized TEBVs after 1 week in culture was similar to values after 5 weeks. TEBVs without endothelium cultured for one week produced significantly less NO in the culture media than that produced in the presence of endothelium (p < 0.05).

After 1 week of perfusion, hNDF TEBVs with and without endothelium constricted after perfusion with 1 μM phenylephrine. Endothelialized hMSC TEBVs perfused for 1 week elicited a smaller contractile response to phenylephrine (Fig. 4d) than TEBVs made with hNDFs. Endothelialized hNDF TEBVs cultured at 2 mL/min for five weeks maintained a steady constriction response to 1 μM phenylephrine (Fig. 4e). TEBVs exhibited a dose response to phenylephrine after 7 days in culture. The magnitude of the response to phenylephrine increased after five weeks in culture, likely due to greater contractile behavior of the medial cells (Fig. 4f).

Acetylcholine elicits endothelium-dependent vasodilation through nitric oxide release in arteries with healthy endothelium. Exposure to 1 μM acetylcholine elicited dilation in endothelialized TEBVs made from either hNDFs or hMSCs cultured for 1 week, and constriction in TEBVs made using hNDFs but without endothelium (Fig. 4g). Endothelialized hNDF TEBVs perfused at 2 mL/min for five weeks maintained a steady vasodilation response to 1μM acetylcholine, indicating sustained endothelial health over time (Fig. 4h). An acetylcholine dose response curve indicates that endothelialized hNDF TEBVs cultured for 1 week tend to plateau in their response at doses above 1 μM acetylcholine. However, endothelialized TEBVs cultured for 5 weeks exhibited increasing vasodilation for acetylcholine doses as high as 100 μM (Fig. 4i). In contrast, TEBVs cultured for one week without endothelium demonstrate a dose-dependent vasoconstriction in response to acetylcholine with a plateau response after 1 μM. Endothelialized TEBVs pre-treated with the nitric oxide synthase inhibitor L-N^G^-Nitroarginine methyl ester (L-NAME) for 10 minutes demonstrated vasoconstriction in response to acetylcholine suggesting that vasodilation was due to release of nitric oxide (Fig. 4i).

### Responses to caffeine and theophylline

**Figure 5:**
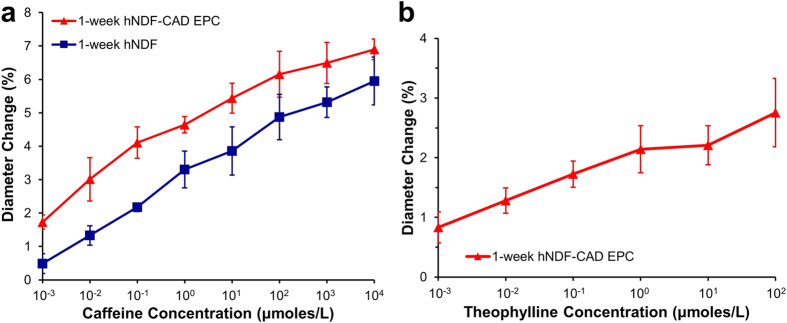
TEBVs perfused for one week elicit dose-dependent vasodilation in response to caffeine (A) and theophylline (B). A two-way repeated measures ANOVA demonstrates a significant effect of dose and the presence of the endothelium in response to caffeine (^#^p < 0.05). Data reported as mean ± SEM, n = 3 TEBVs (**a**). TEBVs exhibit dose-dependent vasodilation in response to theophylline. A one-way repeated measures ANOVA demonstrates a significant effect of dose (P < 0.05). Data reported as mean ± SEM, n = 6 TEBVs tested in three independent experiments (**b**).

Caffeine and theophylline are vasodilators that cause drug-induced vascular injury (DIVI) in several species^33^. Caffeine stimulates NO production in endothelium and inhibits myosin light chain kinase activity on SMCs. Caffeine produces vasodilation of human mammary arteries in the presence of functional and dysfunctional endothelium^34^. TEBVs cultured for one week with and without endothelium exhibited a dose-dependent vasodilation response to caffeine concentrations ranging from 10^−9^ to 10^−2^ M (Fig. 5a) that elicited similar trends to human mammary arteries with functional and dysfunctional endothelium^34^. Theophylline is a less active metabolite of caffeine^34^. Following short-term exposure to theophylline concentrations ranging from 10^−9^ to 10^−4^ M, endothelialized TEBVs cultured for one week exhibited a smaller dose-dependent vasodilation as similar does of caffeine (Fig. 5b). TEBV response to drugs known to cause drug-induced vascular injury provides a promising avenue for studying the effects of this phenomenon *in vitro*.

### Response of TEBVs to TNF-α

**Figure 6:**
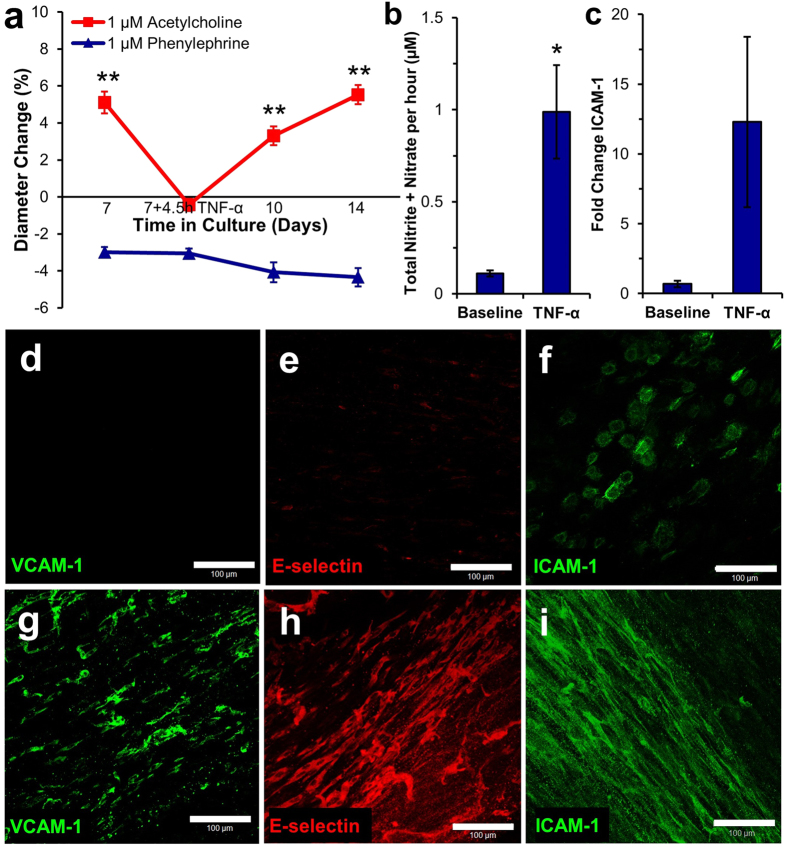
Characterization of acute inflammatory response of TEBV endothelium. Endothelialized TEBVs elicit 5.1 ± 0.6% (mean ± SEM, N = 3 independent experiments. EPCs from 3 CAD donors were used and responses were pooled) dilation in response to 1 μM acetylcholine after 7 days of culture under laminar perfusion; however, after exposure to 200 U/mL TNF-α for 4.5 hours, TEBVs demonstrated an average vasoconstriction of −0.4 ± 0.3%. Three days after the removal of TNF-α, TEBVs demonstrated 3.3 ± 0.5% vasodilation in response to acetylcholine. Seven days after the removal of the TNF-α, TEBVs exhibited normal vasodilation in response to acetylcholine of 5.5 ± 0.5%, indicating that the endothelium is capable of recovering from an acute inflammatory stimulus. A one-way ANOVA indicates a significant effect of treatment day (P < 0.0001). A post-hoc Tukey’s test indicates a statistically significant drop in dilation in response to acetylcholine with TNF-α treatment (**P < 0.005 with respect to Day 7 + 4.5h TNF-α). (**a**). Treatment with 200 U/mL TNF-α for 4.5 hours increased the rate of nitric oxide within the TEBV circuit measured after perfusion for 48 hours (*p < 0.05) (**b**). Treatment with 200 U/mL TNF-α resulted in increased expression of ICAM-1 mRNA in hNDFs (**c**). *En face* images of TEBV endothelium demonstrate limited basal expression of cell adhesion molecules VCAM-1 (**d**), E-selectin (**e**), and ICAM-1 (**f**). Treatment with 200 U/mL TNF-α for 4.5 hours increases the expression of VCAM-1 (**g**), E-selectin (**h**), and ICAM-1 (**i**).

To evaluate an acute inflammatory response, endothelialized TEBVs were perfused for 1 week, then exposed to 200 U/mL TNF-α for 4.5 hours^35^. We examined the response to 1 μM phenylephrine and 1 μM acetylcholine before and up to 7 days after the transient exposure to TNF-α. After exposure to 200 U/mL TNF-α for 4.5 hours, TEBVs constricted after exposure to acetylcholine (Fig. 6a). The endothelium began to recover three days after TNF-α exposure, and 7 days post-exposure, acetylcholine-induced vasodilation was the same as it was before exposure to TNF-α, indicating that the TEBV endothelium recovered from an acute inflammatory stimulus. The contractile response to phenylephrine was unaffected by TNF-α treatment (Fig. 6a).

Treatment with 200 U/mL TNF-α for 4.5 hours caused an increase in the rate of NO production compared to TEBVs under baseline conditions (Fig. 6b). TNF-α increases the amount of induced nitric oxide synthase expression within vascular smooth muscle cells^36^. Exposure to 200 U/mL TNF-α in the flow circuit resulted in increased expression of ICAM-1 mRNA in hNDFs within the vessel wall (Fig. 6c). In the absence of TNF-α, the protein expression of VCAM-1 (Fig. 6d), E-selectin (Fig. 6e), and ICAM-1 (Fig. 6f) is low or absent. *En face* images of the endothelium demonstrated increased expression of VCAM-1, E-selectin, and ICAM-1 after exposure to 200 U/mL TNF-α within the flow circuit for 4.5 hours (Fig. 6g–i), indicating endothelial activation^37^.

### TEBV Response to Lovastatin

**Figure 7:**
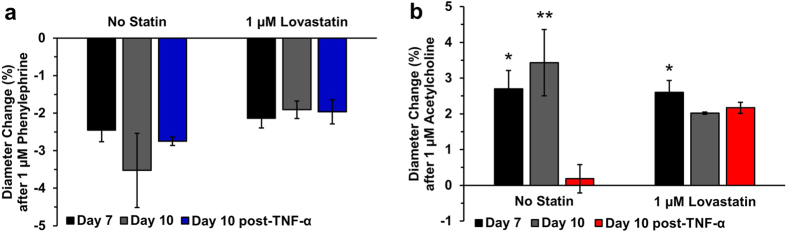
TEBV response to lovastatin, Endothelialized TEBVs cultured for 7 days and then exposed to 1 μM lovastatin for 3 days, or maintained without statin. On Day 10, TEBVs were exposed to 200 U/mL TNF-α for 4.5 hours, then exposed to 1 μM phenylephrine followed by 1 μM acetylcholine. Treatment with lovastatin demonstrated no effect on the endothelium-independent vasoconstriction in response to phenylephrine (**a**). Treatment with lovastatin substantially preserved the endothelium-dependent vasodilation response. A one-way repeated measures ANOVA and a post-hoc Dunnett’s test with respect to Day 10 post-TNF-α showed that while TNF-α treatment reduced vasodilation in the absence of statin (*P < 0.05, **P < 0.005) relative to conditions not exposed to TNF- α or statin, lovastatin treatment blocked the effect of TNF-α on vasodilation (**b).**

Since statins exhibit pleotropic atheroprotective effects on endothelial cells^38^, we examined whether lovastatin treatment of TEBVs could block the response to TNF-α. Endothelialized hNDF-TEBVs were matured for one week, and then exposed to 1 μM lovastatin in the flow circuit for 3 days, or maintained without treatment, after which they were exposed to 200 U/mL TNF-α for 4.5 hours. Exposure to TNF-α or lovastatin had no effect on the endothelium-independent response to phenylephrine (Fig. 7a). While vessels not pre-treated with lovastatin prior to exposure to TNF-α did not undergo vasodilation in response to acetylcholine, exposure to lovastatin sustained vasodilation in response to acetylcholine after exposure to TNF-α on Day 10 (Fig. 7b).

## Discussion

The human TEBVs developed in this study had several novel features. They could be prepared with inner diameters of 500–800 μm and perfused in less than three hours. In contrast, other approaches to prepare TEBVs require 6–8 weeks *in vitro* culture before the mechanical strength is sufficient to enable perfusion^14^,^39^. After 1 week of perfusion, medial hNDFs or hMSCs expressed contractile proteins α-smooth muscle actin and calponin, indicating a switch to a contractile phenotype^31^. TEBVs also produced the extracellular matrix proteins laminin, collagen IV, and fibronectin and exhibited burst pressures similar to human saphenous veins (1599 ± 877 mm Hg)^29^. Quantifiable and physiologically relevant reactions to vasoactive stimuli occurred after only 1 week. TEBVs released nitric oxide, elicited endothelium-independent vasoconstriction to phenylephrine and endothelium-dependent vasodilation in response to acetylcholine, and maintained these responses during 5 weeks of *in vitro* perfusion culture.

Non-destructive monitoring of maturation and function is critical for an effective microphysiological system for drug screening. In this study, vasoactivity was quantified *in situ* to evaluate the TEBV maturation and endothelial health. Prior work, as referenced in Table 1, has evaluated endothelium-independent vasoconstriction of TEBVs in response to serotonin, endothelin-1, and prostaglandin^17^,^40^. The vasoconstriction of TEBVs produced with collagen gels embedded with rat smooth muscle cells has been evaluated destructively through changes in tension upon exposure to endothelin-1, bradykinin, phenylephrine, and sodium nitroprusside^41^. Recently, we evaluated endothelium-independent vasoconstriction in a TEBV after six weeks of fabrication using the cell sheet method^42^. In the current study, monitoring vasoconstriction and vasodilation non-destructively allowed us to evaluate the function of both endothelial cells and medial cells within the TEBVs over time. Most importantly, the TEBVs in this study produced physiological responses to drug stimuli (Table S3), suggesting functional maturation after only 1 week of perfusion at physiological flow rates. Lastly, the raw vasoactive responses of our TEBVs are within the physiological range for human native arteries, since human radial arteries dilate between 3–10%^43^.

An effective microphysiological system must be capable of measuring tissue responses under both healthy and diseased conditions. Endothelial activation and dysfunction affects patients with hypercholesterolemia^44^, diabetes mellitus^45^, and complications from chronic smoking^46^, leading to atherosclerosis^47^. Activated or dysfunctional endothelium exhibit reduced vasodilation in response to acetylcholine^21^. Acute endothelial activation was induced in response to the pro-inflammatory cytokine TNF-α to validate the inflammatory response of our TEBV system. TNF-α promotes expression of cell-surface adhesion molecules such as VCAM-1, ICAM-1, and E-selectin, which facilitate leukocyte adhesion to the vascular wall^48^. TEBVs initially elicited vasoconstriction in response to acetylcholine after exposure to TNF-α, then recovered their vasodilation response to acetylcholine 7 days after removal of the stimulus in the flow circuit, suggesting endothelial activation and dysfunction without apoptotic injury^37^. In contrast, endothelium-independent vasoconstriction in response to phenylephrine remained the same despite treatment with TNF-α. Studies with rat aortas indicate slightly increased endothelium-independent vasoconstriction in response to phenylephrine upon exposure to TNF-α^49^. Lastly, we exposed the TEBVs to 1 μM lovastatin for 3 days prior to exposure to TNF-α, and pretreatment of TEBVs with lovastatin enabled them to maintain endothelium-dependent vasodilation in response to acetylcholine after exposure to TNF-α, while untreated TEBVs experienced significantly reduced dilation. Lovastatin exhibits pleiotropic atheroprotective effects on endothelial cells by upregulating endothelial nitric oxide synthase (eNOS) expression under inflammatory conditions^50^.

We used CAD EPCs to evaluate the responses of a population pre-disposed to endothelial activation. Late-outgrowth EPCs are harvested from adult peripheral blood or umbilical cord blood with a minimally invasive vein puncture and cultured *ex vivo* to yield characteristics of mature endothelium^51^, allowing for the characterization of TEBVs constructed from cells from various donor populations. CAD EPCs behave similarly to endothelium from healthy patients when seeded over synthetic substrates *in vitro*^26^ and in direct co-culture with human aortic smooth muscle cells *in vitro*^27^.

Evaluating drug-induced vasodilation in a human model would be a significant development in the progress toward finding human biomarkers for DIVI. DIVI typically manifests in animal studies through changes in vascular tone, leading to endothelial dysfunction and inflammation^52^. Endothelial dysfunction has been detected in patients who have undergone treatment with cytotoxic drugs for chemotherapy^53^. Caffeine and theophylline are nonspecific phosphodiesterase inhibitors that elicit vasodilation. A previous study noted that human mammary arteries with healthy endothelium demonstrated greater vasodilation in response to caffeine compared to those with dysfunctional endothelium^34^. Our study noted a statistically significant increase in vasodilation in endothelialized TEBVs compared to non-endothelialized TEBVs. TEBVs also elicited a short-term dose-dependent response to theophylline, suggesting that a DIVI response may be measured in this system.

A limitation of our study was that TEBVs were matured under laminar flow using a peristaltic pump, during which only flow rate was controlled. Cyclic mechanical stress through pulsatile flow increases extracellular matrix production and mechanical strength of TEBVs^13^,^14^; however, laminar flow is sufficient to evaluate the effects of drug stimuli on the endothelium. Pressures in our system were lower than those encountered in the arterial systems and raising the pressure may facilitate differentiation.

Ideally, adult SMCs would be used as the cell source for the vessel wall; however, adult SMCs exhibit limited population doublings *in vitro* before undergoing senescence^54^. We performed this study with hNDFs as a proof of concept due to their attainability, contractile properties, and proliferative capacity. We also examined hMSCs, which have been used to create functional TEBVs^55^, and may be differentiated to express characteristic SMC markers. We are currently evaluating the response of our model with SMCs derived from induced pluripotent stem (iPS) cells, which would enable us to examine patient specific functional changes. Overall, this study indicates that rapidly producible dense collagen gel TEBVs have the potential to serve as *in vitro* models for drug testing under healthy and induced inflammatory conditions.

## Materials and Methods

### Cell Isolation and Culture

All isolations of endothelial progenitor cells from blood were performed in accordance with the protocol approved by the Duke University Institutional Review Board for the collection of peripheral blood from consenting patients undergoing left heart catheterization at Duke University Medical Center. Informed consent was obtained from all donors prior to blood withdrawal. CAD EPCs from four donors over the age of 55 with advanced CAD were isolated and grown as previously stated^26^. CAD EPCs were maintained in EC medium comprised of Endothelial Basal Medium (EBM-2) with an EGM-2 Single Quots Kit (Lonza) supplemented with 10% heat-inactivated fetal bovine serum (HI-FBS, Gibco) and 1% Pen/Strep (Lonza). CAD EPCs were passaged at 80% confluence using 0.025% trypsin/EDTA (Lonza) and neutralized with 1:1 ratio of EC medium. Cells in passages 6–9 were used for all experiments.

Human neonatal dermal fibroblasts were purchased from Clonetics and maintained in hNDF medium comprised of Dulbecco’s Modified Eagle’s Medium (DMEM) with 4.5 g/L glucose (Gibco) supplemented with 10% HI-FBS, 1% Pen/Strep, 1 × Non-essential amino acids (NEAA, Gibco), 1 × Sodium Pyruvate (Gibco), 1 × Glutamax (Gibco) and 0.1% β-mercaptoethanol (Gibco). hNDFs were passaged at 80% confluence using 0.05% trypsin/EDTA (Lonza) and neutralized with a 1:1 ratio of hNDF media. Passages 8–12 were used for all experiments. Human bone marrow-derived MSCs used in this study were generously provided by Darwin J. Prockop of Texas A&M Institute for Regenerative Medicine.

TEBVs under static and flow conditions were maintained in culture medium comprised of DMEM with 1.1 g/L glucose, L-glutamine, and 110 mg/L sodium pyruvate (Gibco) supplemented with 3.3% HI-FBS, 1 × NEAA, 1% Pen-Strep, and 0.1% β-mercaptoethanol. After 1 week in culture, 2 mg/mL ε-aminocaproic acid was added to the TEBV medium to minimize TEBV constriction under long-term culture conditions.

### Flow Cytometry

CAD EPCs were characterized using flow cytometry. Cells were positive for endothelial cell markers CD31 and CD144, and negative for CD45, CD115, and CD14. Mouse IgG1 and untreated cells were used as controls. Antibodies (Biolegend) were conjugated with either FITC or phycoerythrin. Detailed antibody information can be found in Supplementary Table S4. CAD EPCs were passaged using 0.025% trypsin/EDTA at 80% confluence. Approximately 500,000 CAD EPCs were resuspended in 100 μL 1% bovine serum albumin buffered with Dulbecco’s PBS with calcium and magnesium. Cells were incubated at room temperature for 30 minutes with 5 μL of preconjugated antibody before washing with 1% BSA solution. Cells were collected after centrifugation at 400 × g for 7 minutes and fixed using 10% neutral buffered formalin before storage at 4 °C until analysis. Flow cytometry analysis was performed by collecting 9,000 events per sample.

### TEBV Fabrication, Endothelialization and Perfusion

TEBVs were fabricated from collagen gels and plastically compressed to increase the collagen fiber density^25^,^56^. Rat tail collagen I (BD Biosciences) was diluted to 2.05 mg/mL using 0.6% acetic acid (Sigma). A serum-free 10 × Dulbecco’s Modified Eagle’s Medium (DMEM) was added at a 1:10 ratio to the collagen (Sigma). The pH was raised to 8.5 using 5M sodium hydroxide (Sigma). A suspension of hNDFs was added at an initial cell density of 5 × 10^5^ cells/mL. Gel solutions were immediately transferred to a 3-cc syringe with a closed two-way luer-lock stopcock (Cole-Parmer) attached. An 810-μm diameter steel mandrel was inserted in the center to create the TEBV lumen and held in place with Parafilm (Kimberly Clark) at the top of the syringe. Solutions were allowed to gel for 30 minutes at room temperature. Immediately afterward, gel solutions were suspended on 10 KimWipes under a 0.8 μm membrane filter (Whatman). After removal of the water, TEBVs were immediately placed in TEBV media until mounting in a chamber.

TEBVs were mounted in custom-made vascular perfusion chambers that could accommodate 1–2 TEBVs on grips (0.760 mm outer diameter) (Fig. 1). TEBVs were sutured in place using 4-0 black silk sutures. Endothelialization was performed by injecting 500,000 CAD EPCs through the lumen of each TEBV, and the chamber was sealed and rotated at 10 rotations per hour (rph) on a custom-made rotation platform for 30 minutes at 37 °C to allow for cell adhesion. A peristaltic pump (Masterflex) with a minicartridge pump head with 8 rollers (Ismatec) was used to create pulseless flow through the TEBVs. Flow circuits accommodating 1 or 2 TEBVs were created using a reservoir and a combination of silicone and PharMed BPT tubing (Cole-Parmer). Circuits contained 35 mL of flow media as described above per TEBV. Media was changed every 2–3 days. TEBVs were maintained at a flow rate of 2 mL/min throughout all experiments.

### Burst Pressure Analysis

TEBVs were maintained in perfusion chambers with one end sealed, and the other end attached to a differential pressure gauge (Keller). TEBVs were filled passively with PBS until failure^14^.

### Determination of TEBV Inner Diameter

The intraluminal pressure drop across the TEBV was found using strain gauge pressure transducers (Model SPR 524, Millar, Houston, TX) flanking the inlet and outlet of the TEBV. Pressure drop was recorded using a custom Labview program as the TEBV was perfused at flow rates ranging from 2–3.5 mL/min. Inlet pressures ranged from 9.5–20 mm Hg. Intraluminal pressure drops ranged from 0.5–5 mm Hg over a 5-week perfusion period. Poiseulle’s law was used to calculate the inner radius (R), where η = 0.083 g/cm-sec as determined by a viscometer, and L = 1 cm.
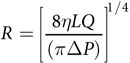


The accuracy of the pressure measurements was corroborated by evaluating various sizes of rigid tubing of known inner diameter in place of the TEBV. Inner diameters of 517 ± 31 μm and 729 ± 53 μm were obtained for tubing of inner diameters of 535 μm and 735 μm, respectively, as measured optically. TEBV outer diameter was evaluated using a stereomicroscope (Amscope) combined with a video camera and imaging software. TEBV wall thickness was evaluated by subtracting the calculated inner diameter from the measured outer diameter.

To assess whether any vessel changes occurred during these measurements, we computed the strain from the Law of Laplace assuming the vessel behaved like an elastic material for small deformations. Based on the Young’s modulus computed from the ultimate tensile stress, the strains ranged between 0.007–0.012, suggesting that the radius change induced by expansion of the vessel wall was between 2.8% and 4.9%.

### Analysis of TEBV Vasoactivity

TEBVs were placed under a stereoscope connected to a video camera (Amscope). Video recordings of the vessel outer diameter were analyzed using Image J. Endothelium-independent vasoconstriction response was evaluated by adding 1 μM phenylephrine (Sigma) to the flow circuit. After 5 minutes, 1 μM acetylcholine (Sigma) was added to the flow circuit to assess the endothelium-dependent vasodilation response^22^. For long-term studies, drug-induced vasoactivity was assessed weekly. A silicone syringe port (Ibidi) was added to the flow circuit and drugs were added to the system via a syringe connected to a sterile 27-gauge needle. Dose-response curves for phenylephrine and acetylcholine were evaluated in TEBVs cultured with and without endothelium for 1 week and with endothelium for 5 weeks. Dose response studies were performed by increasing drug concentration in the flow circuit every 5 minutes. Phenylephrine and acetylcholine dose responses were evaluated using concentrations ranging from 10^−9^ to 10^−4^ M. Caffeine and theophylline (Sigma) dose responses were evaluated using concentrations ranging from 10^−9^ to 10^−2^ M. To evaluate the effect of eNOS inhibition on TEBV vasodilation, L-N^G^-Nitroarginine methyl ester (L-NAME, Sigma) was added to the flow circuit to obtain a final concentration of 3.2 uM and TEBVs were incubated for 10 minutes at 37 C prior to performing an acetylcholine dose-response curve^57^. Changes in outer diameter are expressed as percent change from the baseline outer diameter.

### RNA Isolation and Reverse Transcriptase-Polymerase Chain Reaction Analysis

NCBI reference sequences for the mRNA of each gene were obtained from PubMed, and primer sequences (Table S4) were generated using the online design program Primer-Blast. Primer pairs that demonstrated uniform and single-product melt curves upon reaction with hNDF reference RNA were selected. Primer efficiencies were calculated using standard curves with reference RNA. Primer efficiencies between 90–115% were deemed acceptable.

Cells were extracted from the TEBV matrix by submerging TEBVs in 1 mg/mL collagenase solution in PBS at 37 °C for 30 minutes. TEBVs were spun down at 300 × g for 5 minutes to pellet cells. EPCs and hNDFs were separated using 25 μL CD31 Dynabeads (Life Technologies). Total RNA from hNDFs was isolated using an Aurum Total RNA Mini Kit (BioRad). RNA purity and concentration was assessed using a NanoDrop Spectrophotometer. Reverse transcription of RNA into cDNA was performed using 250 ng of hNDF RNA using the iScript cDNA Synthesis Kit (BioRad). RT-PCR was performed using the iQ SYBR Green Supermix (Bio-Rad) and the CFX Connect Real-Time PCR Detection System (Bio-Rad). Fold change from reference RNA was calculated as previously described^58^. Calponin fold change was normalized to a TEBV cultured for 24 hours under static conditions.

### Immunofluorescent Staining of TEBVs

TEBVs were fixed in 10% formalin for 30 minutes, rinsed three times with DPBS, then cut *en face*. For imaging of contractile proteins and vWF, cells were permeabilized with 0.1% Triton-X in PBS for 5 minutes, and then rinsed three times with DPBS. Blocking was performed with 10% goat serum (Gibco) in DPBS for at least 8 hours at room temperature. Primary antibody staining was performed at 1:100 in 10% goat serum overnight. The following primary antibodies were used: rabbit anti-αSMA, rabbit anti-calponin, rabbit anti-vWF, rabbit anti-laminin, rabbit anti-collagen IV, mouse anti-fibronectin, mouse anti-VCAM-1, mouse anti-ICAM-1, and rabbit anti-E-selectin. All primary antibodies were purchased from Abcam and Santa Cruz Biotechnologies. Detailed antibody information can be found in Supplementary Table S5. After staining overnight with primary antibody, samples were rinsed 3 times with DPBS and stained for 1 hour at room temperature with 1:500 goat anti-rabbit Alexa Fluor 594 or goat anti-mouse Alexa Fluor 488 (Life Technologies). Samples were rinsed 3 times and stained with 1 μL/mL Hoechst 33342 dye in DPBS for 5 minutes at room temperature, then rinsed 3 times with DPBS. Samples were mounted onto slides using Fluor Save reagent (Calbiochem) and covered with a cover slip. Z-stack images of TEBVs were obtained on a Zeiss 510 inverted confocal microscope at 20 × magnification. Images were analyzed using a Zeiss LSM image browser and Image J (NIH).

### Quantification of Nitric Oxide Produced by TEBVs

After 1 week and 5 weeks of culture under perfusion, 5 mL media samples were removed from the flow circuit and maintained at −20 °C. After thawing, proteins were removed using 10,000 MWCO spin columns (Corning) microcentrifuged at 10,000 rpm for 5 minutes. Total nitrite and nitrate in each sample was assessed using a Greiss reagent assay kit (Pierce). Absorbance at 540 nm was measured using a μQuant microplate reader (Bio-Tek). Samples were normalized to pure media controls.

### Effect of TNF-α on TEBVs

Endothelial dysfunction and recovery was evaluated via acute exposure to TNF-α^59^. Endothelialized TEBVs were allowed to mature for 7 days. A 500 μL media sample was collected and frozen at −20 °C for NO analysis. TEBV vasoactivity was evaluated after 7 days using the phenylephrine-acetylcholine assay listed above. TNF-α was introduced to the flow circuit for 4.5 hours at a final concentration of 200 U/mL. TEBV vasoactivity was evaluated after 7 days using the phenylephrine-acetylcholine assay. Media was changed directly afterward to TEBV media with 2 mg/mL ε-aminocaproic acid. TEBV vasoactivity was assessed using the phenylephrine-acetylcholine assay three days and seven days later to evaluate endothelial recovery after removal of the TNF-α stimulus.

The effect of lovastatin toward mediating TEBV response toward TNF-α was evaluated by culturing endothelialized TEBVs made with hNDFs for 1 week. On Day 7, lovastatin (Sigma) was introduced into the flow circuit to produce a final concentration of 1 μM. Control TEBVs were evaluated without exposure to lovastatin. TEBVs were treated for 3 days, and then exposed to 200 U/mL TNF-α for 4.5 hours. TEBV vasoactivity was assessed using the phenylephrine-acetylcholine assay at Day 7, Day 10, and Day 10 after treatment with TNF-α.

### Statistical Analysis

Statistical analyses were performed using JMP 11 (SAS Institute). All data were analyzed by one-way or two-way analysis of variance (ANOVA) with post hoc Tukey’s test to compare means. A one-way repeated measures ANOVA was performed for all time and dose-dependent assays. All data are represented as means ± SEM. *P* values < 0.05 were considered significant. Power calculations were performed to ensure sample sizes yielded a power greater than 0.8.

## Additional Information

**How to cite this article**: Fernandez, C. E. *et al*. Human Vascular Microphysiological System for *in vitro* Drug Screening. *Sci. Rep.* **6**, 21579; doi: 10.1038/srep21579 (2016).

## Supplementary Material

Supplementary Information

## Figures and Tables

**Table 1:** TEBV Vasoactivity.

**Tissue Type**	**Endothelium?**	**Vasoactive Agent added to Medium**	**Assessment Method**	**Ref.**
TEBV comprised of human umbilical vein derived SMCs	No	Multiple doses: Endothelin-1, Endothelin-2	Destructive; Isometric force measurements of TEBV rings.	40
Rat aortic SMCs in collagen gel mold	No	Multiple doses: Endothelin-1, Bradykinin, Phenylephrine, BHT-920, ATP disodium salt, 5-hydroxytryptamine	Destructive; Isometric force measurements of TEBV rings.	41
Bovine SMCs cultured on biodegradable PGA scaffold	Yes; bovine ECs	Multiple doses: Serotonin, Endothelin-1, Prostaglandin	Destructive; Isometric force measurements of TEBV rings.	17
Silastic tubing embedded within the peritoneal cavity of rats	Yes; non-thrombotic mesothelial cells lining intima	Single doses: KCl, Acetylcholine, Phenylephrine	Destructive; Isometric force measurements of TEBV rings.	60
Nanopatterned human bone-marrow derived MSCs grown on a scaffold-free matrix	Yes; human cord-blood derived EPCs	Single dose; Phenylephrine	Non-destructive; changes in TEBV diameter analyzed under perfusion as a result of exposure to vasoactive agents	42
Human NDFs or bone-marrow derived MSCs embedded in a dense collagen gel matrix	Yes; adult peripheral blood EPCs	Multiple doses: Phenylephrine, Acetylcholine, Caffeine, Theophylline	Non-destructive; changes in TEBV diameter analyzed under perfusion as a result of exposure to vasoactive agents under varying doses.	Current study
