# Supplementary material for: Human Vascular Microphysiological System for in vitro Drug Screening
Source: Sci Rep. 2016 Feb 18;6:21579. doi: 10.1038/srep21579 (PMC4757887; doi:10.1038/srep21579)
Supplement: Supplementary Information [file srep21579-s1.pdf]

Human Vascular Microphysiological System for *in vitro* Drug Screening

Supplementary Information

Authors:

C. E. Fernandez[1], R. W. Yen[1], S. M. Perez[1], H. W. Bedell[1], T. J. Povsic[2], William M. Reichert[1], G. A. Truskey[1*]

Author Affiliations:

[1]Department of Biomedical Engineering, Duke University, Durham, NC 27708

[2]Duke Clinical Research Institute, Duke University Medical Center, Durham, NC 27708

*To whom correspondence should be addressed: gtruskey@duke.edu

Supplementary Materials and Methods:

S1. Tensile Testing of TEBVs

Circumferential tensile strength was analyzed using a micro-strain analyzer (TA Instruments) with modified grips as shown in Fig. S1. TEBVs were cut in 5 mm sections and mounted through grips with diameters of 300 μm. Cyclic pre-conditioning was performed by stretching the lumen to a strain of 20% for 6 cycles (*1*). An optimized strain rate of 0.08 mm/s was used to stretch rings until failure.

Grip diameters were taken into account in calculating the strain (ε), where D = grip diameter. Stress (σ) was calculated by assuming conservation of volume.

$$\varepsilon = \frac{L_i - L_s}{L_s};\ L_s = 2D + L_0$$

$$\sigma = \frac{F}{A}\,(1 + \frac{\Delta L}{L_0})$$

Ultimate tensile stress was defined as the maximum stress before failure. The Young's modulus (E) was calculated by dividing stress by strain.

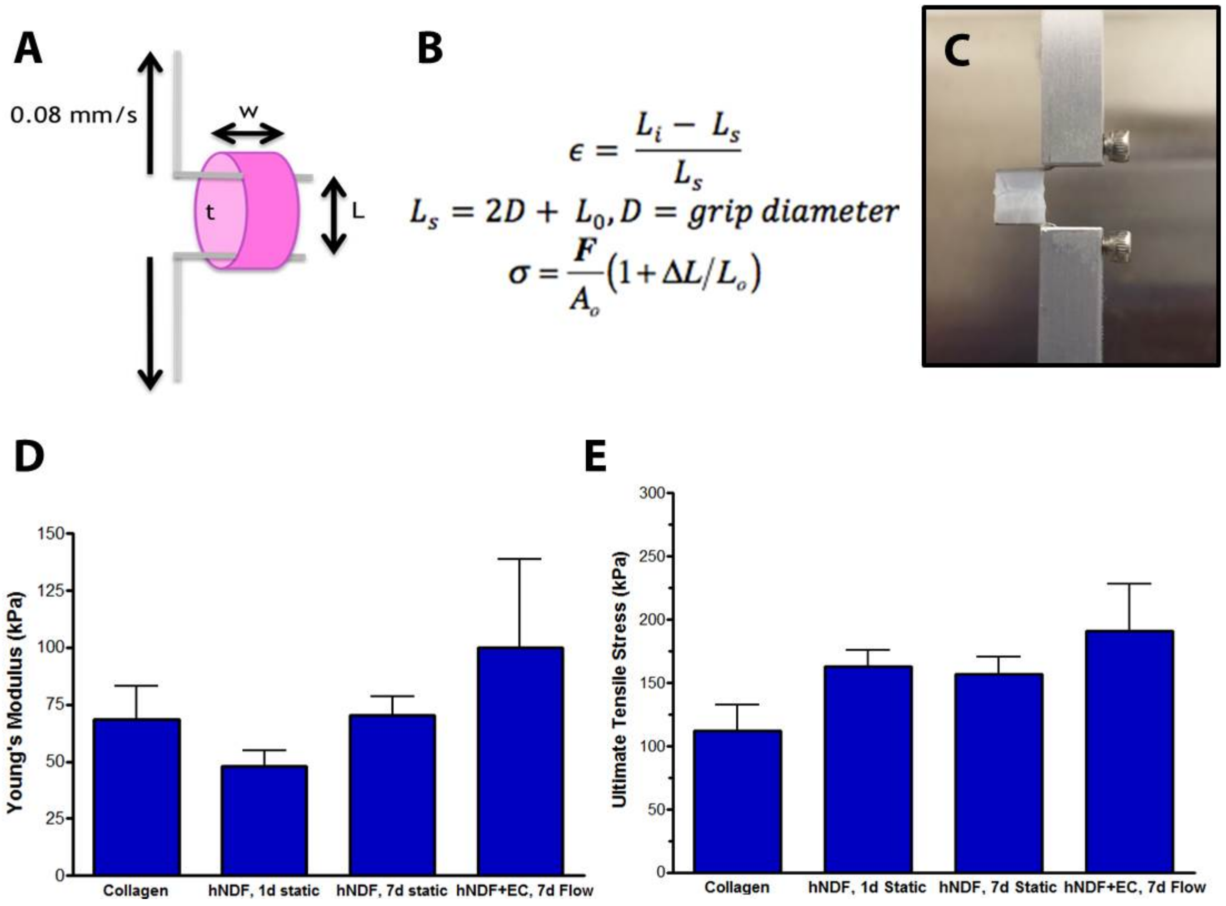

Fig S1: TEBV Mechanical Testing. TEBVs were cut into rings approximately 5 mm in width (w) and mounted on modified grips (**A**). Stress and strain were calculated by taking the diameter change into account as the TEBV was pulled in tension (**B**). TEBVs stretched significantly throughout the mechanical test (**C**). Mechanical stimulation had a demonstrated impact on TEBV Young's modulus (**D**), and ultimate tensile stress (**E**). Data shown as mean ± SEM, n = 3-7 TEBVs.

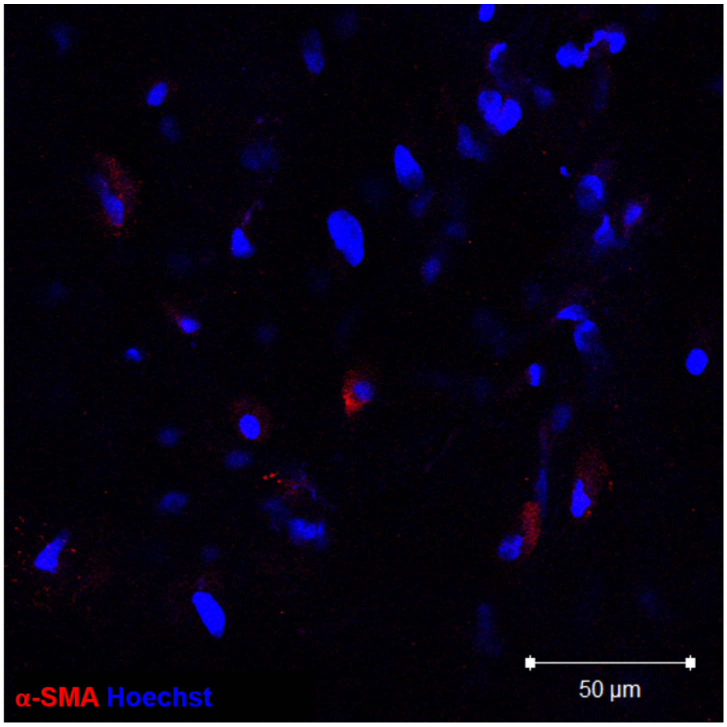

Fig. S2: Expression of α-SMA in hNDF TEBVs after 24 hours of culture.

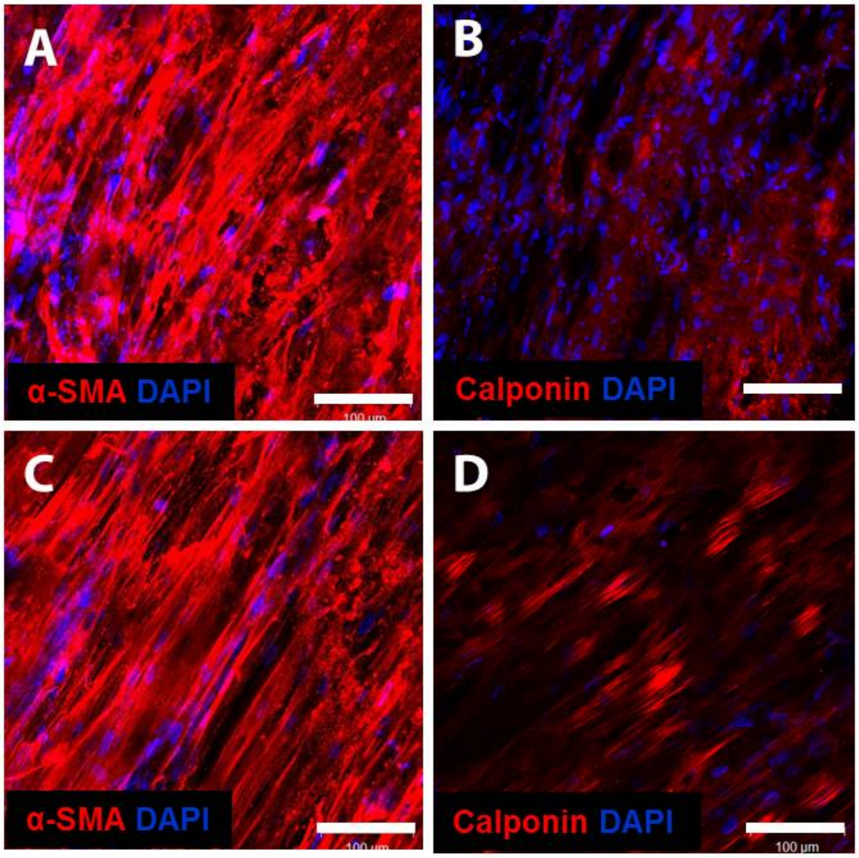

Fig S3: Contractile protein expression of endothelialized TEBVs made with hNDFs or hMSCs matured for 5 weeks under perfusion. TEBVs made with hNDFs express α-SMA (**A**) and calponin (**B**). TEBVs made with hMSCs substantially increase production of α-SMA (**C**) and calponin (**D**) during 5 weeks of perfusion at physiological flow rates. All scale bars indicate 100 µm.

Table S1: TEBV Collagen Fiber Density

Initial CFD (%)	Water Loss (%)	Final CFD (%)	Fold Increase in Collagen Density
0.23 ± 0.01	96.2 ± 0.8	6.2 ± 1.2	26.1 ± 5.9

Table S2: Primers for RT-qPCR.

Target (Gene)	Primer Sequence
ICAM-1 (ICAM1)	Fwd: 5’-CAC CCT AGA GCC AAG GTG AC-3’<br>Rev: 5’- GGG CCA TAC AGG ACA CGA AG-3’
α-SMA (ACTA2)	Fwd: 5’-GAC CTT TGG CTT GGC TTG TC-3’<br>Rev: 5’-GTG CGG ACA GGA ATT GAA GC-3’
Calponin1 (CNN1)	Fwd: 5’-AGG TTA AGA ACA AGC TGG CCC-3’<br>Rev: 5’-ATG AAG TTG TTG CCG ATG CG-3’
B2-microglobulin (B2M)	Fwd: 5’-GGC TAT CCA GCG TAC TCC AAA G-3’<br>Rev: 5’-CAA CTT CAA TGT CGG ATG GAT G-3’

Table S3: Human Vasoactivity Responses

Drug	Target	EC_{50}	Reference
Phenylephrine	Human saphenous vein	10^{-5} M	(*2*)
Acetylcholine	Human brachial artery	0.537×10^{-6} M	(*3*)
Theophylline	Human dorsal hand vein	84 µg/min	(*4*)
Caffeine	Human mammary artery	10^{-6} M	(*5*)

Table S4: Antibodies for Flow Cytometry

Antigen	Conjugate	Source	Isotype	Vendor	Clone
CD31	FITC	Mouse	IgG1, κ	BioLegend	WM59
CD144	PE	Mouse	IgG2a, κ	BioLegend	BV9
CD14	FITC	Mouse	IgG1, κ	BioLegend	HCD14
CD45	FITC	Mouse	IgG1, κ	BioLegend	HI30
CD115	PE	Rat	IgG1, κ	BioLegend	9-4D2-1E4
Mouse IgG1	FITC	Rat	IgG	BioLegend	RMG1-1

Table S5: Antibodies for Immunofluorescence

Type	Antigen	Conjugate	Source	Clonality	Isotype	Vendor	Number
1°	vWF	--	Rabbit	Polyclonal	IgG	Abcam	ab6994
1°	α-SMA	--	Rabbit	Polyclonal	IgG	Abcam	ab5694
1°	Calponin	--	Rabbit	Monoclonal	IgG	Abcam	ab46794
1°	Fibronectin	--	Mouse	Monoclonal	IgG1	Abcam	ab26245
1°	Collagen IV	--	Rabbit	Polyclonal	IgG	Abcam	ab6586
1°	Laminin	--	Rabbit	Polyclonal	IgG	Abcam	ab91006
1°	VCAM-1	--	Mouse	Monoclonal	IgG1	Santa Cruz	sc-13160
1°	E-selectin	--	Rabbit	Polyclonal	IgG	Santa Cruz	sc-14011
1°	ICAM-1	--	Mouse	Monoclonal	IgG1	Santa Cruz	sc-107
1°	Rabbit IgG	--	Rabbit	--	IgG	Life Technologies	02-6102
2°	Mouse IgG	Alexa Fluor 488	Goat	Polyclonal	IgG	Life Technologies	A-11001
2°	Rabbit IgG	Alexa Fluor 594	Goat	Polyclonal	IgG	Life Technologies	A-11012

Supplementary References

1. E. D. Grassl, T. R. Oegema,R. T. Tranquillo, A fibrin-based arterial media equivalent. *J Biomed Mater Res A* **66**, 550-561 (2003).
2. C. M. Crowley, C. H. Lee, S. A. Gin, A. M. Keep, R. C. Cook,C. Van Breemen, The mechanism of excitation-contraction coupling in phenylephrine-stimulated human saphenous vein. *Am J Physiol Heart Circ Physiol* **283**, H1271-1281 (2002).
3. T. A. Bruning, M. G. Hendriks, P. C. Chang, E. A. Kuypers,P. A. van Zwieten, In vivo characterization of vasodilating muscarinic-receptor subtypes in humans. *Circ Res* **74**, 912-919 (1994).
4. M. Grossmann, J. Braune, U. Ebert,W. Kirch, Dilatory effects of phosphodiesterase inhibitors on human hand veins in vivo. *Eur J Clin Pharmacol* **54**, 35-39 (1998).
5. D. Echeverri, F. R. Montes, M. Cabrera, A. Galan,A. Prieto, Caffeine's vascular mechanisms of action. *Int J Vasc Med* **2010**, 834060 (2010).
